# Supplementary figures and images for: Mental health of health care workers during and after the COVID-19 pandemic – A longitudinal cohort study
Source: PLOS Ment Health. 2025 Jun 11;2(6):e0000333. doi: 10.1371/journal.pmen.0000333 (PMC12798477; doi:10.1371/journal.pmen.0000333)

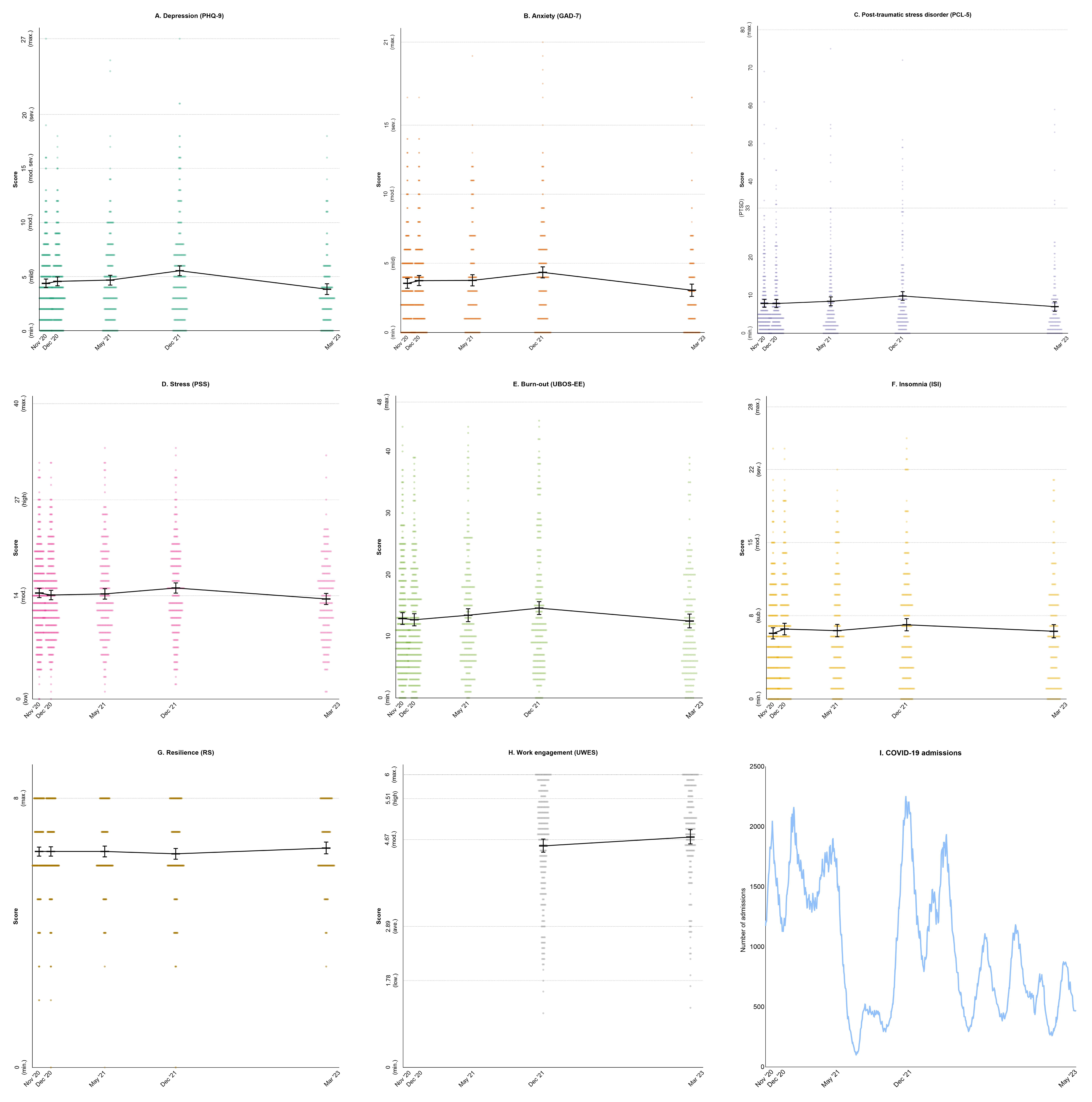

Supplement: S1 Fig — ure A-I: On the x-axis the timeline is presented in days since the start of this study (November 2020). A-H: Means and 95% confidence intervals (CIs) of the survey sum scores are depicted per time point by small black horizontal lines and black vertical lines, obtained from the univariable model. A: On the y-axis sum scores per individual (teal dots) of symptoms of depression (assessed by PHQ-9) are presented, the dotted grey lines represent the different clinical cut-offs scores. B: On the y-axis sum scores per individual (orange dots) of symptoms of anxiety (assessed by GAD-7) are presented, the dotted grey lines represent the different clinical cut-offs scores. C: On the y-axis sum scores per individual (purple dots) of symptoms of post-traumatic stress disorder (assessed by the PCL-5) are presented, the dotted grey line represent the clinical cut-offs score. D: On the y-axis sum scores per individual (pink dots) of perceived stress (assessed by PSS), the dotted grey lines represent the different clinical cut-offs scores. E: On the y-axis sum scores per individual (green dots) of symptoms of burn-out (assessed by UBOS-EE) are presented. F: On the y-axis sum scores per individual (yellow dots) on symptoms of insomnia (assessed by ISI) are presented, the dotted grey lines represent the different clinical cut-offs scores. G: On the y-axis sum scores per individual (brown dots) of resilience (assessed by RS) are presented. H: On the y-axis sum scores per individual (grey dots) of work engagement (assessed by UWES) are presented, the dotted grey lines represent the different clinical cut-offs scores. I: Publicly available data from the national coordination center for patient distribution in The Netherlands (‘Landelijk coördinatiecentrum patiënten spreiding’) was used to depict the number of COVID-19 in-hospital admitted patients nationwide (continuous blue line) per day during study follow-up [28]. Abbreviations: min. = minimum sum score; mod. = moderate sum sc [file pmen.0000333.s009.tif]

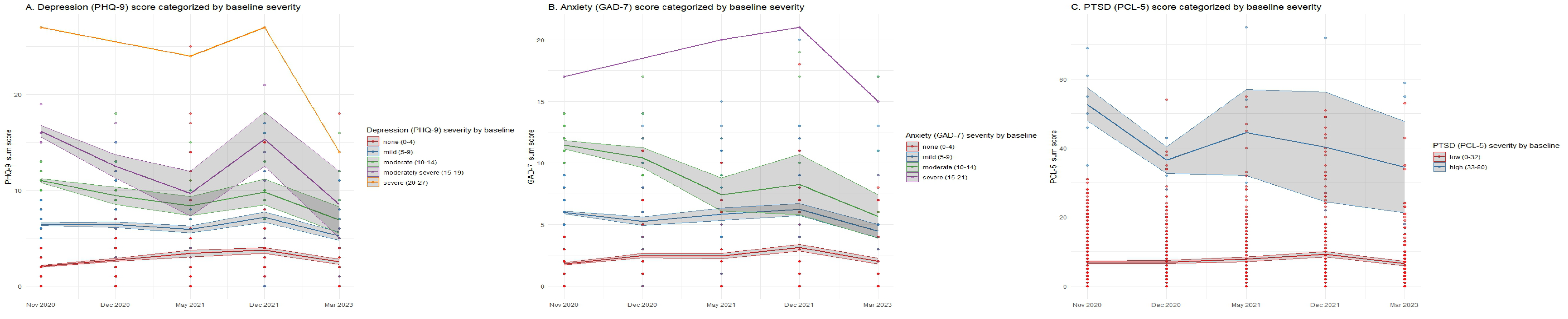

Supplement: S2 Fig — Participants were categorized by clinical determined cut-off scores indicating the severity of depression (PHQ-9), anxiety (GAD-7) and post-traumatic stress disorder (PTSD [PCL-5]), based on sum scores in November 2020. Individual data points, plotted with dots, indicate the distribution of PHQ-9, GAD-7 and PCL-5 sum scores at each time point. The mean scores for each severity category (based on November 2020) are represented by solid lines, with shaded areas around the lines depicting the standard error. The X-axis represents the different time points of data collection. The Y-axis indicate the sum scores, with higher scores reflecting greater severity of depression, anxiety and PTSD. (TIF) [file pmen.0000333.s010.tif]

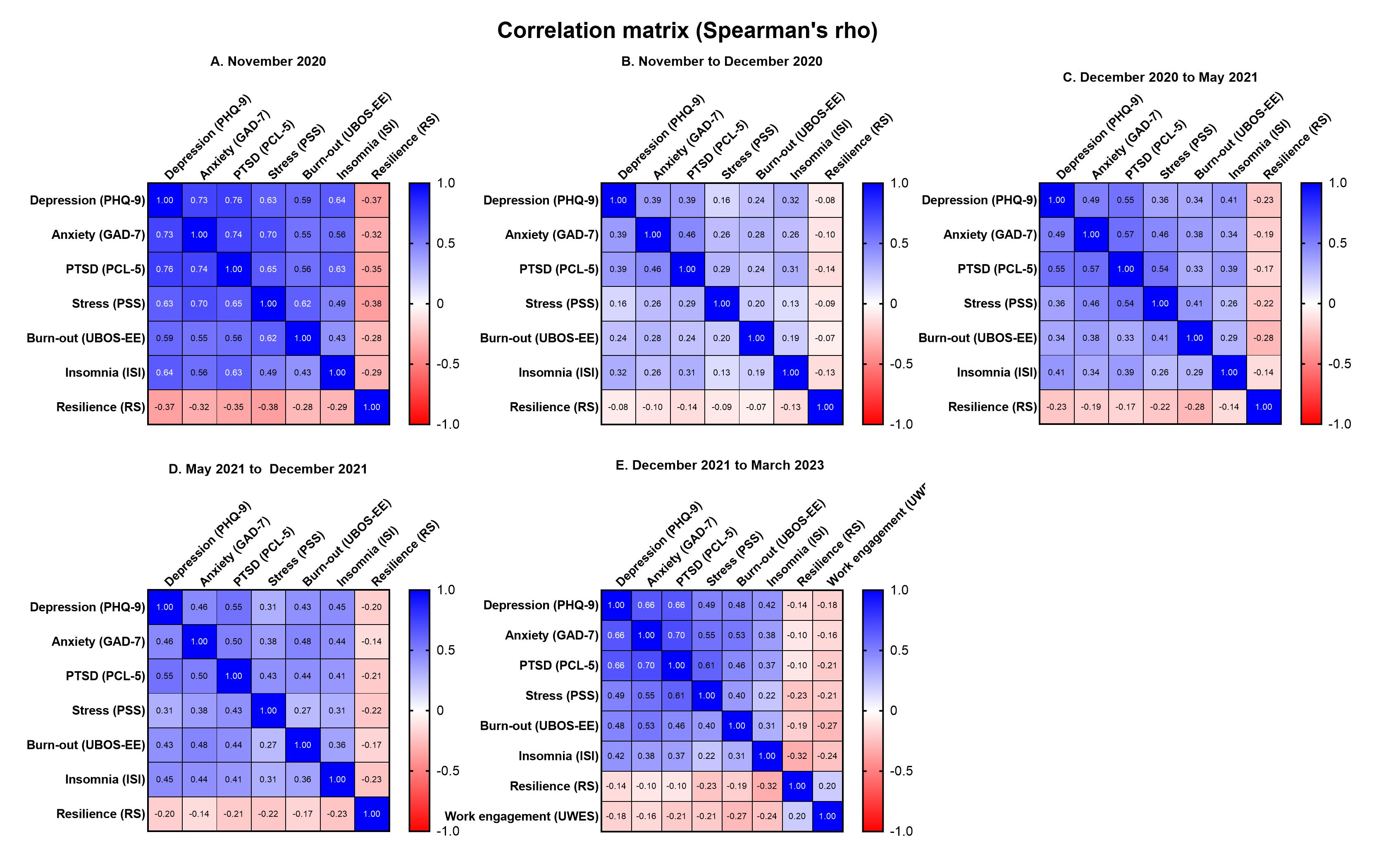

Supplement: S3 Fig — Deltas of individual sum scores were calculated between follow-up time points for each participants. Spearman’s rank correlation coefficient was used to assess the degree of correlation between the deltas of the different survey outcomes. Higher sum scores on the depression (PHQ-9), anxiety (GAD-7), post-traumatic stress disorder (PTSD [PCL-5]), stress (PSS), burn-out (UBOS-EE) and insomnia (ISI) surveys indicated decreased mental health. Higher sum scores on the resilience (RS) and work engagement (UWES) surveys indicate better resilience and work engagement. (TIF) [file pmen.0000333.s011.tif]
