# Supplementary material for: Mental health of health care workers during and after the COVID-19 pandemic – A longitudinal cohort study
Source: PLOS Ment Health. 2025 Jun 11;2(6):e0000333. doi: 10.1371/journal.pmen.0000333 (PMC12798477; doi:10.1371/journal.pmen.0000333)
Supplement: Attachment — Submitted filename: ResponseReviewersAndEditor20250115.docx [file pmen.0000333.s013.docx]

**Point by point response to editor and reviewers**

Reviewer's Responses to Questions

**Comments to the Author**
1. Does this manuscript meet PLOS Mental Health’s [publication criteria](https://eur04.safelinks.protection.outlook.com/?url=https%3A%2F%2Ftrack.editorialmanager.com%2FCL0%2Fhttps%3A%252F%252Fjournals.plos.org%252Fmentalhealth%252Fs%252Fcriteria-for-publication%2F1%2F010f019386158bdf-8b928148-e5ac-467e-8d59-25a7d3672ea3-000000%2FNC0wTQM0tW2YssRgus_VElN6zW3-nL0-pF-ISZhoEY4%3D187&data=05%7C02%7Ca.lavell%40amsterdamumc.nl%7Cac3368c729b1411a11be08dd129ba21b%7C68dfab1a11bb4cc6beb528d756984fb6%7C0%7C0%7C638687181831934291%7CUnknown%7CTWFpbGZsb3d8eyJFbXB0eU1hcGkiOnRydWUsIlYiOiIwLjAuMDAwMCIsIlAiOiJXaW4zMiIsIkFOIjoiTWFpbCIsIldUIjoyfQ%3D%3D%7C0%7C%7C%7C&sdata=t5TiSRnPFA3YED%2FZd3kzgOjWbd53nwb0%2Bda6fmbMsUQ%3D&reserved=0)? Is the manuscript technically sound, and do the data support the conclusions? The manuscript must describe methodologically and ethically rigorous research with conclusions that are appropriately drawn based on the data presented.

Reviewer #1: Yes

Reviewer #2: Partly

*Answer: We clarified the used methodology and presentation of the results in an improved version of our manuscript.*

2. Has the statistical analysis been performed appropriately and rigorously?

Reviewer #1: Yes

Reviewer #2: No

*Answer: We addressed this concern in the following section, in response to the detailed comments of reviewer #2.*

3. Have the authors made all data underlying the findings in their manuscript fully available (please refer to the Data Availability Statement at the start of the manuscript PDF file)?
The [PLOS Data policy](https://eur04.safelinks.protection.outlook.com/?url=https%3A%2F%2Ftrack.editorialmanager.com%2FCL0%2Fhttps%3A%252F%252Fjournals.plos.org%252Fmentalhealth%252Fs%252Fdata-availability%2F1%2F010f019386158bdf-8b928148-e5ac-467e-8d59-25a7d3672ea3-000000%2FQyEi5ChVccT74n-oGPuP1TCPvfIk1Kcpu82_DNHiquk%3D187&data=05%7C02%7Ca.lavell%40amsterdamumc.nl%7Cac3368c729b1411a11be08dd129ba21b%7C68dfab1a11bb4cc6beb528d756984fb6%7C0%7C0%7C638687181831957991%7CUnknown%7CTWFpbGZsb3d8eyJFbXB0eU1hcGkiOnRydWUsIlYiOiIwLjAuMDAwMCIsIlAiOiJXaW4zMiIsIkFOIjoiTWFpbCIsIldUIjoyfQ%3D%3D%7C0%7C%7C%7C&sdata=g6wFa0y%2Bb%2BKPYZN6shJvBZYHckw%2FkrA%2BApfgYLgfXXM%3D&reserved=0) requires authors to make all data underlying the findings described in their manuscript fully available without restriction, with rare exception. The data should be provided as part of the manuscript or its supporting information, or deposited to a public repository. For example, in addition to summary statistics, the data points behind means, medians and variance measures should be available. If there are restrictions on publicly sharing data—e.g. participant privacy or use of data from a third party—those must be specified.

Reviewer #1: Yes

Reviewer #2: Yes

4. Is the manuscript presented in an intelligible fashion and written in standard English?
PLOS Mental Health does not copyedit accepted manuscripts, so the language in submitted articles must be clear, correct, and unambiguous. Any typographical or grammatical errors should be corrected at revision, so please note any specific errors here.

Reviewer #1: Yes

Reviewer #2: Yes

5. Review Comments to the Author
Reviewer #1: I find this to be a very useful study which helps establish the effects of COVID-19 on the mental health of workers providing mental health services. The article is well written and the data is well presented.

Reviewer #2: I would like to thank the authors for the opportunity to read this paper. Mental health of HCWs is often neglected. I would like to commend the authors for trying to address this important issue in health, especially at the back of the COVID-19 pandemic where these important professionals were at risk of contracting COVID and carrying heavier workloads, among other workplace and societal ills. Insight into mental health dynamics and determinants in HCWs during the pandemic could help to maintain and improve mental health and resilience in future pandemics. Despite the merit, I feel that this manuscript could be improved before being considered by the journal. There seems to be a mismatch between the objective and the methodology - I think could the methodology be improved. For meaningful descriptive statistics, I suggest using regression sample. This will do away with varying sample sizes. I have attached my detailed comments.

*Answer: We would like to thank both reviewers for their comprehensive review of our work and seeing the value of our manuscript. We will address the concerns of reviewer #2 concerning the used methodology in the following section. In short, the adjustments we made consists of an overall clarification of the used methodology and presentation of the results.*

**Detailed comments of reviewer #2**

**However:**

Not claiming knowledge monopoly, I have reservations on the methodological approach used, especially on the estimation techniques. In repeated cross-sectional surveys, panel data, or cohort studies, using a balanced sample is the best way especially when the goal is to compare outcomes at different time points. This helps in dealing with the potential biases that can emanate from missing observations. For example, it could be that HCWs who dropped out of other surveys were out because of mental health issues. Also, from your writing, I was expecting a FE estimation/regression given that you have demographic variables, - with your outcome variables and continuous dependent variables. As it stands, your estimations are difficult to follow, too many tables – which could have been avoided by simple focused analyses. Simple correlations and comparisons of outcomes without controlling for unobservables just don’t suffice.

For meaningful descriptive statistics, I suggest using regression sample. This will do away with varying sample sizes. Changes in sample sizes can bias your results.

*Answer: We thank Reviewer #2 for their attention to our analysis and their feedback. We do however disagree with the statement that a balanced sample is the best way to account for longitudinal data with dependent repeated measures in our study, including missing data. The current state of the art in statistics to account for the problems arising with such data is to use sophisticated regression models such as mixed models, which we used. As we do take all reviewer feedback very seriously, we checked this with the leading statistician in our centre on this subject, prof dr. J Twisk. He confirmed that the linear mixed model was the most appropriate choice for our data, given its ability to handle unbalanced data and account for both fixed and random effects. However, he suggested as well a more clear presentation of the results and used analysis techniques.*

*To answer the other concerns raised by the reviewer: our linear mixed model incorporates a fixed effect for the time point of the survey and a random intercept for each individual, allowing us to account for individual differences in baseline levels and changes over time. In addition, we included potential confounders (e.g., baseline demographics) as fixed effects in the multivariable model to adjust for their influence on the outcomes.*

*We agree that a balanced sample may have been suitable when data are missing completely at random (MCAR). However, in our study, the missingness is possibly related to observed and unobserved variables (e.g., participants dropping out due to mental health issues or participants leaving the institution). Excluding participants with missing data to create a balanced sample could lead to selection bias and reduce the effective sample size, potentially compromising the generalizability of our findings.*

*.*

*Following the reviewer’s suggestions, we have updated our manuscript to:*

- *Clarify the analytical techniques used in the study.*
- *Streamline the presentation of results, including consolidating tables and focusing on key findings, to enhance readability and comprehension.*

*We are grateful for the reviewer’s input, which has contributed to improving the quality and clarity of our work.*

**As a general comment:** Please work on the captions of **all figures and tables** (please). It’s neat to insert captions above the table and use the space below for notes – which should be written in smaller font to differentiate the from content. I don’t understand these inconsistencies – sometimes you caption once, and sometimes twice, notes and captions combined, etc.

*Answer: Thank you for pointing out these inconsistencies. We have corrected them in the revised manuscript. We have combined Tables 2 and 3 for greater clarity in the representation of results and moved them to the supplement, as Figures 1 and 2 already present the same results and numbers. Additionally, Figure 2 has now been integrated into Figure 1 as panel 1J.*

**Specific comments raised by the reviewer #2:**

**Abstract**

**Line 44** can be clearer/neater if you do away with the second brackets – just combine the information into one sentence: for example, using “with”.

*Answer: we rephrased this sentence into “A total of 384 HCWs (95% of HCWs given consent) completed at least one survey, with 326 (81%) completing two or more surveys.”*

**Introduction**

**Line 69:** *“Less is known about HCWs mental health trends during the COVID-19 pandemic and what happened during its aftermath.”* What exactly do you mean here because in the previous paragraph you have provided evidence that HCWs mental health declined.

*Answer: Thank you for your comment. In the previous section, we provided evidence of a decline in mental health after the first wave of the pandemic. In this sentence, we aim to highlight the limited research on longitudinal mental health trends of HCWs during the subsequent pandemic years and in the immediate aftermath. We have rephrased the sentence as follows: “Less is known about how HCWs' mental health evolved during the COVID-19 pandemic and in the immediate aftermath.”*

**Methods**

***Data collection and outcomes***

***Primary outcomes***

**Line 115-120:** the range of scores showed that you transformed the responses to the mental health domains – but you didn’t highlight that. I also know that you will say the likert scales start at 0, but why? For lay people with interests in mental health – you should indicate what you did/ or why.

*Answer: Thanks for raising this unclarity. This depends on the questionnaire we are using and how we compare our scores with scores from other studies. If the reference studies also started counting Likert scales with 0, we followed their example to make comparison clearer and hopefully understandable for reviewers and readers. We have now revised the methods section to briefly address this and have removed detailed references to the numbers to avoid any potential confusion.*

***Secondary outcomes***

**Line 131:** does resilience fall under secondary outcomes? From the title of your paper its is a primary variable.

*Answer: We are grateful that you raise this inconsistency. As stated in our preregistration, resilience was measured as our secondary outcome. We therefore removed this term from out title and changed this into “Mental health of health care workers during and after the COVID-19 pandemic – a longitudinal cohort study”.*

**Results**

***Study population***

**Line 175:** The statement on median age does not support what is shown in Table 1.

*Answer: Thank you for pointing that out. It appears that we inadvertently reversed the ages of those working in patient care with those not working in patient care. This statement has now been corrected.*

**Tabel 1:** I don’t understand the importance/rationale of including missing observations. What do they help you? Especially when interpreting your results and suggesting policy?

*Answer: By specifying the number of missing data, we have aimed to be transparent, and avoid any misinterpretation that the cumulative amounts don’t add up.*

**Line 181:** It’s better to insert table caption above the table, and use the space below the table for **notes** only.

*Answer: Thank you for the suggestion. We have made the correction by placing the table caption above the table and using the space below for notes only, as recommended.*

**Longitudinal mental health measurement in HCWs**

***Primary outcomes***

Based on the interpretation of the scores/scales, the mean scores over time suggest that mental health wasn’t an issue at all time points – what do you have to say about mean scores way below midpoints of the scales?

*Answer:* *We appreciate your comment regarding the interpretation of the mean scores, which were notably below the midpoints of the scales. We agree that the mean scores in our cohort of healthcare workers (HCWs) were generally low, and we have emphasized this in the discussion section of our manuscript. Specifically, on page 15 of the discussion section, we state: “Despite the worsening of symptoms during the pandemic, mean scores on symptoms of depression, anxiety and PTSD were notably low in our cohort. In November 2020, 7.9% and 5.9% of HCWs were above the clinical cut-off values for depression and anxiety disorders, respectively. In comparison, the Netherlands Mental Health Survey and Incidence Studies (NEMESIS) reported a 9.8% prevalence for mood disorders and 15.2% for anxiety disorders in the general population between November 2019 and March 2022, with no differences in prevalence before and during the pandemic. Although it is important to note that different diagnostic instruments were used [41]. Some countries reported much higher rates, such as 41% depression, 50% anxiety, and 66% PTSD in Italy [25], highlighting variability between countries, cultures and health care systems [42,43].” We hypothesize that this may partly be explained by our findings that work-related factors appeared to have a limited impact on mental health in our cohort, while non-work-related factors, such as nationwide measures, may have influenced mental health to a greater extent. We also recognize a potential limitation of our study: individuals who were absent, potentially due to experiencing decreased mental health after the first wave pandemic wave, were unable to participate due to the requirement for written informed consent. This may have resulted in selection bias,* *potentially underestimating the overall mental health burden. We have now addressed this limitation more explicitly in the manuscript and discussed its possible impact on our findings.*

**Primary outcomes #2:**

There is not difference in the statistics between November and December 2020 – why did you include both instead of one?

*Answer: As shown in figure 1I, there was a significant surge in COVID-19-related admissions during this period. We believe it is valuable to include both measures taken before and immediately after this surge to provide a comprehensive view of its impact. Additionally, not all participants completed assessments in both November and December 2020. Excluding one of these time points would result in unnecessary data loss.*

**Table 2:**

Please insert caption above table.

*Answer: We have inserted the captions above table 2 and all other tables.*

What does the first row mean?

*Answer: The first row has been excluded for clarity. It originally provided information about the data, such as the mean values with 95% confidence intervals and Cohen’s d, which have now been incorporated into the left side as part of the row names.*

What sample are you using to calculate the statistics?

*Answer: Our whole cohort of HCWs was used to calculate the statistics in table 2. This information has now been included in the subscription of the table.*

**Figure 1**: In most cases, when CIs overlap the difference between the data points is not statistically significant – you might need to consider lightly using the term “significant(ly)”.

*Answer: To clarify, we only consider an effect to be statistically significant if the confidence interval of the difference does not include zero, as outlined in our methods section and presented in Table 2. If the point estimate lies outside the confidence interval, this indicates statistical significance. However, we acknowledge that the figure presented in the manuscript is quite small, and enlarging it would improve visibility.*

**Line 213:** Is Figure 1. As complete sentence?

*Answer: We have corrected all inconsistencies in titles and subscript of tables and figures.*

**Correlation between mental health outcomes**

**Line 243-244:** How is 0.70 a weaker corr when in the previous line 0.73 was considered strong?

*Answer: Thank you for highlighting this inconsistency. As correlation values are often context-dependent and their interpretation can be subjective, we have revised the text to describe the correlations as positive or negative, including the numerical values, without attributing additional interpretive weight to them.*

**Discussion**

There is no introduction to this important section. I expect to be reminded of the objective/aim of the study here.

*Answer: We have now incorporated this sentence at the beginning of the Discussion section: “The aim of this longitudinal cohort study is to gain deeper insight into the course of mental health symptoms among healthcare workers (HCWs) during the peak of the COVID-19 pandemic and its aftermath, with a focus on trends over time and potential determinants.”*

**Line 318:** *… pandemic restrictions despite vaccine availability, which may have caused* ***demoralization*** *and a sense of…* Be consistent of the language you are using (American/British/etc English).

*Answer: Thank you for pointing that out. We have reviewed the manuscript and ensured that it follows American English throughout.*
